# Drastic Cardiac Reverse Remodeling Following Catheter Ablation in Patients with Atrial Fibrillation and Heart Failure

**DOI:** 10.3390/medicina57050511

**Published:** 2021-05-20

**Authors:** Takahisa Koi, Naoya Kataoka, Teruhiko Imamura, Koichiro Kinugawa

**Affiliations:** The Second Department of Internal Medicine, University of Toyama, 2630 Sugitani, Toyama 930-0194, Japan; takahisa.koi@i.softbank.jp (T.K.); nkataoka@icloud.com (N.K.); kinugawa-tky@umin.ac.jp (K.K.)

**Keywords:** hemodynamics, reverse remodeling, arrhythmia

## Abstract

In the management of atrial fibrillation in patients with heart failure, rate control is recommended, whereas the implication of rhythm control remains controversial. We experienced a 65-year-old man who had compensated heart failure due to hypertensive heart disease and atrial fibrillation with well-controlled heart rate (<100 bpm). At three months following the catheter ablation procedure, the left ventricular ejection fraction improved from 40% up to 65%. The implication of rhythm control using catheter ablation in improving cardiac reverse remodeling should be validated in large-scale clinical studies.

## 1. Introduction

There is a considerable association between atrial fibrillation and heart failure. The presence of one increases the likelihood of another [1]. Atrial fibrillation is an independent predictor of mortality in patients with heart failure [2]. 

Atrial fibrillation facilitates the progression of heart failure [3]. The incremental heart rate is associated with tachycardia-induced cardiomyopathy. Tachycardia or bradycardia decreases cardiac output and deteriorates hemodynamics. The loss of atrial kick diminishes left ventricular filling. The existence of atrial fibrillation activates the renin-angiotensin-aldosterone system. 

The first-line therapy for those with heart failure and atrial fibrillation is a rate control using beta-blocker in combination with other medications if necessary [4]. The rhythm control is recommended in selected patients, using antiarrhythmic medications or catheter ablation [5]. However, its impact on cardiac function remains unknown. We experienced a heart failure patient who achieved drastic cardiac reverse remodeling following catheter ablation to treat atrial fibrillation. 

## 2. Care Report

### 2.1. Before Admission

A 65-year-old man with a history of two times of hospitalizations due to worsening heart failure and long-standing untreated hypertension admitted to our institute for further treatment. 

At his first decompensated heart failure, bisoprolol and amiodarone were initiated for his systolic heart failure (45% of left ventricular ejection fraction) and sustained ventricular tachycardia. Following the discharge, persistent atrial fibrillation developed. 

At his second decompensated heart failure, the etiology was diagnosed as hypertensive heart disease according to the findings of endomyocardial biopsy with no myocardial fibrosis and heterogeneous myocardium and 25% of left ventricular ejection fraction as well as no significant coronary artery stenosis demonstrated by coronary angiography. His heart rate was controlled below 100 bpm during the observational period. 

### 2.2. On Admission

His heart failure was relatively compensated with New York Heart Association functional class II using 100 mg of amiodarone, 5 mg of enalapril, 5.0 mg of bisoprolol, 25 mg of spironolactone, 60 mg of azosemide, and 20 mg of furosemide which was added at the second decompensated heart failure re-admission thoroughly. 2.5 mg of warfarin also administrated. Body height was 166 cm and body weight was 65 kg (body mass index 23.7). Blood pressure was 100/74 mmHg and pulse rate was 86 bpm (irregular). An institutional review board approved the method (R2015154, 11 April 2016) and an informed consent was obtained from the patient. 

**Figure 1:**
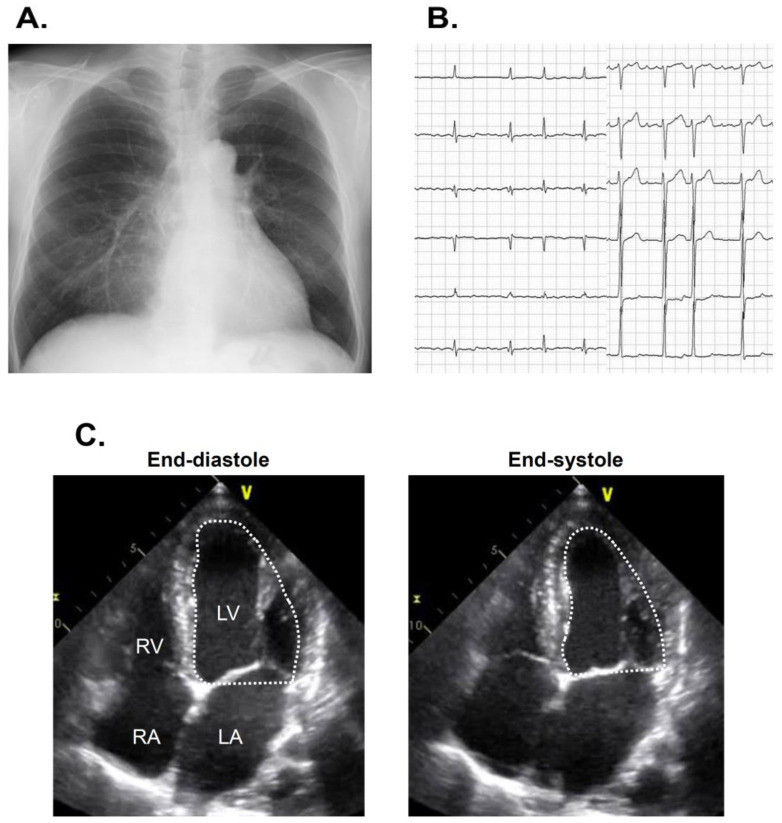
Chest X-ray showing 47% of cardiothoracic ratio (**A**), electrocardiogram showing atrial fibrillation (**B**), and transthoracic echocardiography showing reduced left ventricular ejection fraction on admission (**C**) on admission. LA, left atrium: LV, left ventricle; Ao, ascending aorta; RA, right atrium; RV, right ventricle.

The estimated glomerular filtration ratio was 57 mL/min/1.73 m^2^, plasma B-type natriuretic peptide was 72 pg/mL, and hemoglobin A1c was 6.7%. Chest X-ray showed 46% of cardiothoracic ratio without obvious pulmonary congestion (Figure 1A). An electrocardiogram showed 91 bpm with atrial fibrillation (Figure 1B). Transthoracic echocardiography showed 50 mm of left ventricular end-diastolic diameter, 44 mm of left atrial diameter, and 40% of left ventricular ejection fraction calculated using a modified Simpson’s method (Figure 1C). 

### 2.3. In-Hospital Course

**Figure 2:**
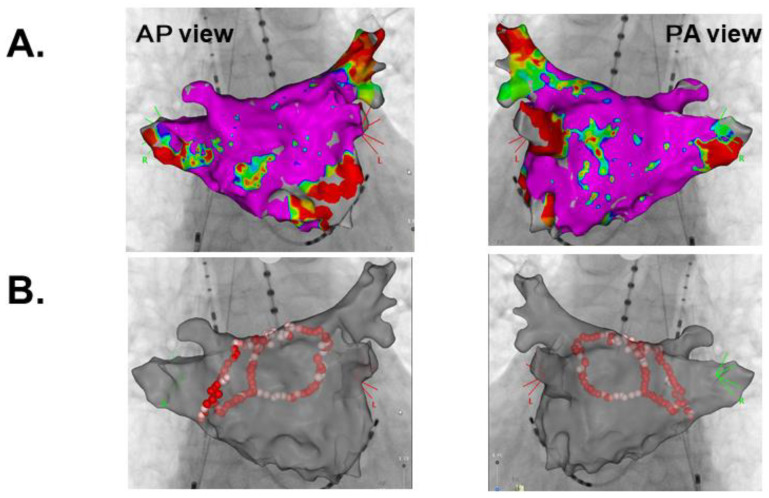
Left atrial voltage mapping (**A**) and ablation lines (**B**). AP, Anterior Posterior; PA, Posterior Anterior.

**Figure 3:**
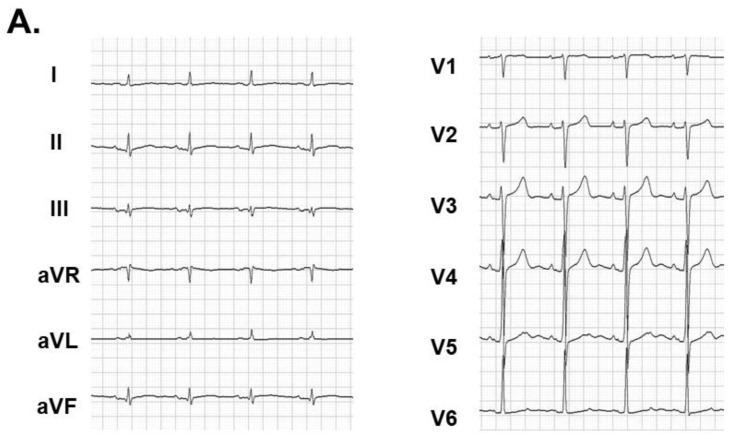

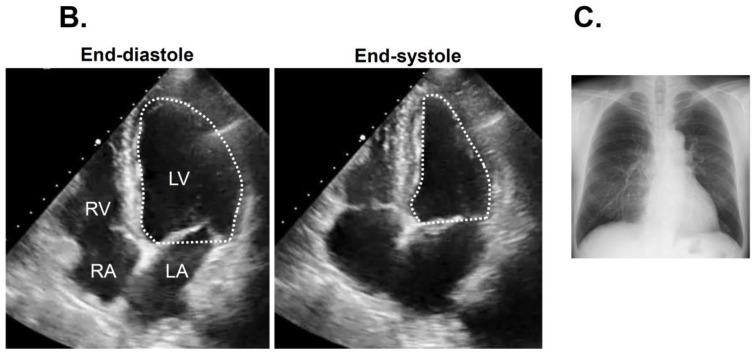
Electrocardiogram at index discharge showing sinus rhythm (**A**). Transthoracic echocardiography showing 65% of left ventricular ejection fraction (**B**) and chest X-ray with 45% of cardiothoracic ratio (**C**) obtained at three months following catheter ablation.

Catheter ablation for persistent atrial fibrillation under general anesthesia was performed using the 3-dimensional mapping system (CARTO 3, Biosense Webster, Diamond Bar, CA, USA). Left atrial voltage mapping using a multipolar catheter (PentaRay, Biosense Webster, Irvine, CA, USA) showed healthy electrical voltage except for in the antrum of left inferior pulmonary vein and left atrial septum (Figure 2A). First, the circular electrical isolation of left pulmonary veins was performed. Next, the posterior wall electrical isolation was completed with the ablation for the right pulmonary veins bottom, anterior, and roof lines (Figure 2B). Following the catheter ablation procedure, the patient was discharged on day 5 with a recovery to sinus rhythm (Figure 3A). 

### 2.4. Three-Month Follow-Up

Following the index discharge, anti-heart failure agents remained unchanged. The post-procedural course was uneventful without any adverse events including the recurrence of atrial fibrillation. The patient was evaluated for AF recurrence by a clinical interview, electrocardiogram, and portable electrocardiograph (OMRON Corp., Kyoto, Japan) every month. New York Heart Association functional class was I with 21 pg/mL of plasma B-type natriuretic peptide at three-month follow-up. Transthoracic echocardiography showed 41 mm of left atrial diameter and 53 mm of left ventricular end-diastolic diameter (Figure 3B). Of note, left ventricular ejection fraction improved up to 65% without any wall motion asynergy. Chest X-ray showed 45% of cardiothoracic ratio (Figure 3C).

## 3. Discussion

### 3.1. Atrial Fibrillation and Heart Failure

Heart failure increases both preload and afterload of the left atrium and progresses its’ remodeling, causing the development of atrial fibrillation [1]. The existence of atrial fibrillation reduces ventricular filling due to the lack of atrial kick and stimulates the renin-angiotensin-aldosterone system despite heart rate is well controlled [3]. In this patient, as heart failure progresses, atrial fibrillation occurred. The new-onset atrial fibrillation might have triggered the second decompensated heart failure episode. 

### 3.2. Indication of Rhythm Control

The first-line to manage atrial fibrillation in patients with heart failure is rate control [4]. If hemodynamics is unstable due to uncontrolled heart rate, electrical cardioversion is considered. Heart rate was well controlled in our patient using a maximum dose of bisoprolol. Given his well-control heart rate, tachycardia-induced cardiomyopathy was excluded. The patient repeated decompensated heart failure against rate control therapy, and we decided to perform rhythm control. 

The Atrial Fibrillation and Congestive Heart Failure (AF-CHF) trial was the first randomized trial that compared the prognostic implication of rhythm control with drug therapy versus rate control (with beta-blockers) in patients with atrial fibrillation and systolic heart failure [6]. The rhythm control strategy did not have a statistically significant advantage over the rate control strategy in improving cardiovascular death. Given the finding, rhythm control using medication is not routinely recommended in patients with atrial fibrillation and heart failure. 

Recently, the Cather Ablation for Atrial Fibrillation with Heart Failure (CASTLE-AF) trial demonstrated the superiority of catheter ablation over the conventional medical therapy for rate or rhythm control in improving all-cause mortality and worsening heart failure in selected patients with atrial fibrillation and systolic heart failure [5]. Given a recent onset of atrial fibrillation and less remodeled left atrium in our patient as well as recent cumulating evidence, we performed catheter ablation. 

### 3.3. Implication of Rhythm Control on Cardiac Reverse Remodeling

Our patient experienced a drastic improvement in left ventricular ejection fraction (approximately 25% increase). Given that guideline-directed medical therapy could not prevent his repeated heart failure recurrences, a dominant cause of his cardiac reverse remodeling would be AF ablation. Such an extreme cardiac reverse remodeling would improve patients’ quality of life and exercise capacity as well as preventing recurrent heart failure. His left ventricular end-diastolic diameter slightly enlarged after AF ablation, probably because of the prolonged diastolic duration by decreased heart rate with converted sinus rhythm. Several small studies also found an approximately 10% increase in left ventricular ejection fraction [7]. The detailed mechanism warrants further studies, but the normalized atrial kick might have reduced the required potential energy. Amelioration of atrial fibrillation might have deactivated the renin-angiotensin-aldosterone system. 

Further analyses are required for optimal patient selection [5]. Shorter heart failure duration, non-ischemic etiology with a less myocardial scar, no recurrence of atrial fibrillation, like our patient, might be keys to improvement in cardiac function following catheter ablation. Less left atrial fibrosis, indicated by the existence of healthy electrical voltage in the whole left atrial field in our patient, would also be another key to the incremental left atrial kick that contributes left ventricular reverse remodeling [8]. Cardiac magnetic resonance image analysis, although we did not perform it, would be useful to quantify the left atrial fibrosis and seek optimal patients. Although accurate detection of AF recurrence needs continuous monitoring for heart rhythm, our patient did not receive cardiovascular implantable electronic device implantation, leading to the limitation of this study [9]. Obesity is known as a risk factor of AF recurrence after ablation, but the patient‘s body mass index was not so high [10]. Glycemic homeostasis and insulin resistance that we did not evaluate may have affected ventricular reverse remodeling [11]. 

## 4. Conclusions

In conclusion, a rhythm control of atrial fibrillation using catheter ablation would achieve drastic cardiac reverse remodeling in carefully selected patients with systolic heart failure. 

## Data Availability

Data are available from the corresponding author upon reasonable request.
